# Moderator Effect of Hypoalbuminemia in Volume Resuscitation and Plasma Expansion with Intravenous Albumin Solution

**DOI:** 10.3390/ijms232214175

**Published:** 2022-11-16

**Authors:** Christian J. Wiedermann

**Affiliations:** 1 Institute of General Practice and Public Health, Claudiana—College of Health Professions, 39100 Bolzano, Italy; christian.wiedermann@am-mg.claudiana.bz.it; 2 Department of Public Health, Medical Decision Making and HTA, University of Health Sciences, Medical Informatics and Technology-Tyrol, 6060 Hall in Tyrol, Austria

**Keywords:** biomarker, colloid osmotic pressure, colloids, crystalloids, fluid resuscitation, human albumin solution, hypoalbuminemia, intervention

## Abstract

Intravenous administration of crystalloid or colloid solutions is the most common intervention for correcting hypovolemia in intensive care unit patients. In critical illness, especially sepsis and severe trauma, vascular wall permeability increases, and trans-endothelial escape of serum albumin, the major oncotic plasma constituent, contributes to the development of hypoalbuminemia and edema formation. The volume effects of intravenous human albumin solution exceed those of crystalloid solutions. If hypoalbuminemia is an effect moderator, the crystalloid-to-albumin ratio of fluid resuscitation volumes is not well characterized. Randomized controlled trials have confirmed that intravenous administration of human albumin solutions for volume resuscitation results in a lower net fluid balance compared with crystalloids, and smaller infusion volumes may be sufficient for hemodynamic stabilization when human albumin solutions are used. This narrative review summarizes the current evidence and conclusions drawn regarding the role of hypoalbuminemia in volume resuscitation. In the ‘Saline versus Albumin Fluid Evaluation’ study using 4% human albumin solution or saline, the saline-to-albumin ratio of study fluids was significantly higher in patients with baseline serum albumin concentrations of 25 g/L or less as compared to patients with baseline serum albumin concentrations of more than 25 g/L. In patients receiving renal replacement therapy, intravenous administration of 20–25% human albumin solution reduces intradialytic hypotension and improves fluid removal better than saline if serum albumin levels are similarly reduced. These data suggest that hypoalbuminemia acts as an effect moderator in volume resuscitation and plasma expansion with albumin solution. The volume effectiveness of intravenous human albumin solution in resuscitation appears to be greater when the serum albumin levels are low. In clinical situations, serum albumin concentrations per se may inform when and how to include intravenous albumin in fluid resuscitation if large amounts of crystalloids are needed, which requires further studies.

## 1. Introduction

Hypovolemia is the leading cause of hemodynamic instability and microvascular perfusion defects in critically ill patients and is often the reason for intensive care monitoring and treatment. Intravenous (IV) administration of infusion solutions is the most common interventions used to correct hypovolemia, especially in intensive care units [1].

The available IV solutions differ in their physiological properties, which are related to their clinical efficacy, frequency, and type of adverse reactions. IV solutions are either crystalloids (e.g., 0.9% saline or lactated Ringer’s solution) or colloids (e.g., hydroxyethyl starch (HES), gelatin, or albumin). The efficacy and safety of fluid administration in hypovolemia are influenced not only by the type of IV solution but also by the amount administered [2]. In hypovolemia, the lack of effective intravascular volume is inadequately compensated for by IV infusion, and perfusion deficits in various organ systems proliferate and worsen. Conversely, an excess of delivered volume also leads to the worsening of clinical outcomes due to edema formation and the consequences of hypervolemia [3].

**Figure 1:**
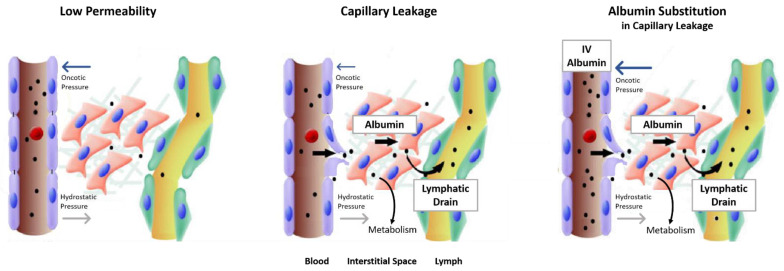
Albumin flux during various conditions. The size of the arrows reflects the direction and sizes of pressures and albumin fluxes. Adapted with permission from Ref. [4]. Copyright © 2022, Springer Nature.

In critical illness, especially sepsis and severe trauma, vascular wall permeability increases, and trans-endothelial escape of serum albumin, the major oncotic plasma constituent, contributes to the development of hypoalbuminemia and edema formation [4]. Increasing plasma volume with IV infusion aims to improve tissue perfusion in a sustained manner; however, significant fluid shifts between the physiological compartments may occur, particularly with repeated fluid administration. IV albumin is the only colloid solution that treats intravascular volume deficits by both the fluid bolus effect and by raising the plasma oncotic pressure of serum albumin (Figure 1).

This narrative review focuses on volume resuscitation and plasma expansion with human albumin solutions and analyzes the potential role of hypoalbuminemia as a moderator of the relationship between IV albumin administration and volume requirement in the fluid management of hypovolemic critically ill patients.

## 2. Evidence-Based Fluid Management of Hypovolemia in Critically Ill Patients

The development of crystalloid infusion therapy dates back to the 19th Century. Colloidal solutions were added later, first with concentrated albumin from human plasma, followed by semi-synthetic artificial colloids [5].

IV infusions for fluid replacement and the maintenance of fluid balance are limited to crystalloid solutions. However, boluses of both crystalloid and colloid solutions are often used in combination for resuscitation and correction of hemodynamic instability [2]. The duration of the achieved expansion of plasma volume by IV solutions is crucial for the sustained improvement of tissue perfusion with reduced risks of edema formation and cumulative positive fluid balance. The duration of the volume-expanding effect of IV solutions is determined by hydrostatic pressure, oncotic pressure, and the semi-permeability of the vessel wall, for which the endothelial glycocalyx plays a crucial regulatory role [6]. 

In addition to maximizing the intravascular effect of administered infusion volumes, fluid management aims to improve the pumping capacity of the heart, which depends on the preload and contractility. For resuscitation in hemodynamic instability, bolus administrations of so-called balanced crystalloids (with a chloride content lower than 0.9% saline) are preferentially used unless there is traumatic brain injury or a reason for decreased serum levels of sodium and chloride to be treated with 0.9% or even hypertonic saline solution [2]. Studies in healthy volunteers have shown that large volumes of lactated Ringer’s solution administered to healthy humans produced small transient changes in serum osmolality. Large volumes of sodium chloride did not change osmolality but resulted in lower pH [7]. A comparison of calculated osmolarity and measured in-vitro osmolality suggests that 4% human albumin solutions, Hartmann’s solution, and, to a lesser extent, gelatine preparations are hypo-osmolar, and may, therefore, increase brain volume and intracranial pressure [8]. In general, colloidal solutions have longer intravascular retention times than crystalloid solutions. Because not only too little but also too much fluid administration is harmful, all IV solutions are considered drugs. In patients at risk of hypervolemia, low-volume resuscitation strategies utilizing the physiological properties of colloid solutions have been developed more recently [9].

### 2.1. Safety Concerns with Semi-Synthetic Colloids

- Pharmaco-epidemiological studies on colloid solutions (HES, gelatin, dextran, and albumin) have shown that, until recently, the use of the semi-synthetic artificial colloids HES and gelatin was many times more frequent than the use of the natural colloid albumin, and overall, colloid was administered to more patients and during more episodes than crystalloid [10]. However, patterns of IV fluid resuscitation, which differed markedly across countries [10], changed because of longstanding controversial safety concerns that are well established and evident but still, practice significantly varied between geographical regions [11]. In light of the increasingly proven safety problems with HES solutions, this artificial colloid has issued warnings and has not been approved for use in intensive care units in recent years. Against this background, and in view of the lack of proof of efficacy for hard clinical endpoints in the indications used, the European Medicines Agency decided in 2022 to withdraw marketing authorization for HES-containing IV solutions. After a short transition period, HES solutions will no longer be available in the European Union [12].
- Dextran solutions are no longer recommended for fluid management because of their numerous adverse effects [13].
- Because of the existing safety concerns with HES- and dextran-based colloids, it is not surprising that gelatin, which has been poorly studied, is being investigated for its safety [14]. A randomized controlled trial (RCT) on the use of gelatin in septic shock was initiated recently [15] but terminated prematurely by the Data Safety and Monitoring Board [16]. It is likely that the potential side effects of gelatin (kidney injury, bleeding, and allergy) are responsible for the early cessation of the RCT.

### 2.2. Safety Concerns with Human Albumin Solutions 

After decades of clinical use as plasma expanders and efforts by hospital pharmacies to better regulate the use of albumin because of its high cost in many hospitals and countries [17], in a 1998 meta-analysis, albumin solutions were evaluated by one of the Cochrane Injuries Group Albumin Reviewers as an intervention potentially leading to increased mortality in patients with hypovolemia, burns, or hypoalbuminemia [18]. Despite justified criticism due to methodological deficiencies of the meta-analysis [19] and accompanied by the intensive promotion of artificial colloids as a cheaper alternative, albumin was increasingly replaced by gelatin and especially so-called “newer generation” HES solutions adapted towards lower molecular weights and substitutions [20,21]. Ultimately, it took a large, double-blind RCT in critically ill patients, the ‘Saline versus Albumin Fluid Evaluation’ (SAFE) study [22], to invalidate the safety concerns from the Cochrane analysis.

**Figure 2:**
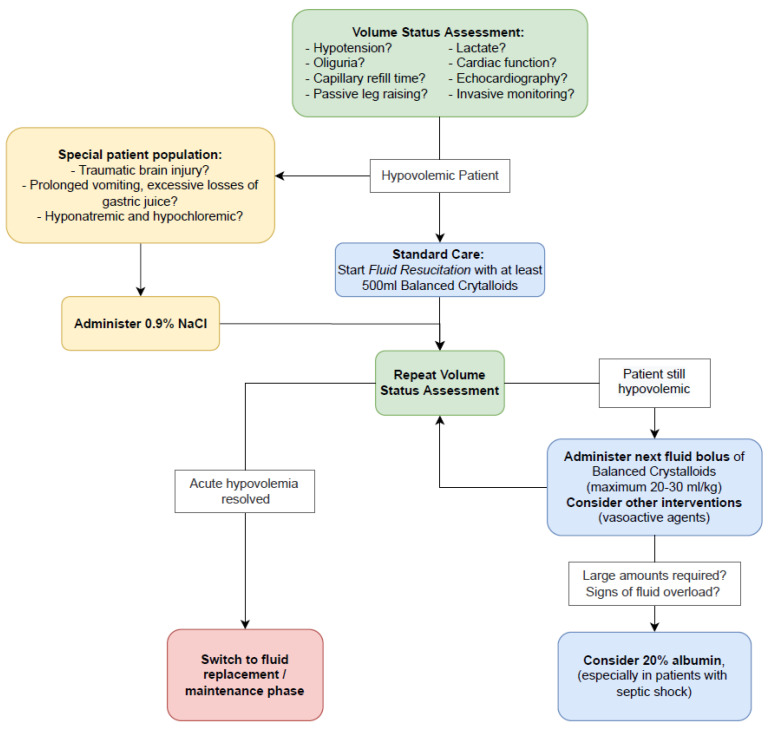
Main considerations for fluid resuscitation in hypovolemic critically ill patients. Reprinted with permission from Ref. [2]. Copyright © 2022, Oxford University Press.

Albumin solutions have a sufficiently good safety profile to remain the only recommended colloid for use in volume resuscitation [23]. According to the Surviving Sepsis Campaign Guidelines for the Treatment of Sepsis and Septic Shock [24] and other Sepsis Guidelines [25,26], crystalloids are the first choice, and albumin is recommended as a safe addition when crystalloids alone are insufficient. Except in situations of pathologically low serum sodium and chloride levels and traumatic brain injury, balanced crystalloid solutions are the preferred IV solutions. In the state of distributive shock due to reduced systemic vascular resistance and impaired oxygen extraction, the longer the phase of hypotension lasts, the worse the treatment outcome. Individual patient factors influence the relationship between arterial blood pressure and organ perfusion and function depending on age and history of chronic arterial hypertension. A mean arterial pressure (MAP) of 65 to 70 mmHg should be adequate for most patients and can usually be achieved in vasopressor-dependent shock by additional administration of norepinephrine followed by combination vasopressor therapies if the MAP goal cannot be reached [24]. The use of 20% human albumin solution in the treatment of hypovolemia is recommended when increased fluid volumes appear necessary for circulatory stabilization, especially when patients already show signs of fluid overload (Figure 2) [2]. This albumin recommendation is based on the superior volume effect of IV albumin compared to IV crystalloids [27].

## 3. Hypoalbuminemia as Effect Moderator of Resuscitation Fluids

**Figure 3:**
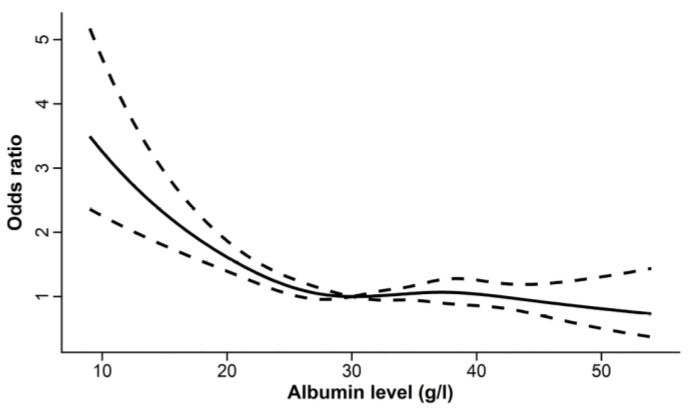
The relationship between serum albumin level and the risk of ICU mortality assessed by restricted cubic splines in a retrospective cohort study of 18,353 patients from the Medical Information Mart for Intensive Care IV (MIMIC-IV) database. The odds ratio of mortality is shown on the *y*-axis. The risk of mortality increases progressively when initial serum albumin levels are lower than 30 g/L. Reproduced without modification from Jin et al. 2022 [31] (accessed on 22 October 2022), under a Creative Commons Attribution 4.0 International (CC BY) License (https://creativecommons.org/licenses/by/4.0/ accessed on 22 October 2022). Copyright © 2022, The Authors. This reuse has not been endorsed by the licensor. The source reference is “[31]” and is available at https://www.frontiersin.org/articles/10.3389/fnut.2022.770674/full, accessed on 13 November 2022.

Decreased serum albumin levels are common in critically ill patients, not only because of trans-capillary redistribution to extra-vascular fluid compartments and intravascular dilution due to administration of IV solutions without albumin but also because of greater losses, metabolization, and decreased synthesis [27,28,29]. Hypoalbuminemia is associated with worsened disease course and treatment outcomes in many acute and chronic inflammatory conditions [28,30]. This association applies to ICU patients and has been confirmed in observational studies on large numbers of patients; however, the sensitivity and specificity for predicting outcomes in critically ill patients are low. A cut-off level of serum albumin <30 g/L has been identified in the association of hypoalbuminemia and increased ICU (Figure 3) and hospital mortalities of ICU patients [31].

The greater volume effects of IV albumin as a colloid compared to crystalloids have been put forward in the crystalloid–colloid controversy based on observational and controlled clinical studies for decades [3,32]. For example, in a propensity score-matched observational study of sepsis patients at increased risk because of cardiac comorbidity, the use of IV albumin was shown to be safe and the day 28 all-cause mortality was significantly reduced in the albumin-receiving group, but the day 90 all-cause and cardiac mortality were not; baseline levels of serum albumin (mean ± standard deviation) in the albumin and control groups were 29.4 ± 5.7 and 29.9 ± 4.6 g/L, respectively [33]. However, systematic studies in RCTs with a low risk of bias have only occurred recently.

The greater volume effects of 4% IV albumin compared to saline, with IV albumin concentration similar to plasma, were confirmed in the SAFE study [22]. If volume resuscitation is performed with 20% IV albumin solution instead of 4–5% IV albumin, then the required infusion volume can be reduced even further. This was demonstrated in ‘A Pilot, Randomized, Unblinded, Feasibility, Safety and Biochemical and Physiological Efficacy Study of 20% vs. 4% Human Albumin Solution for Fluid Bolus Therapy in Critically Ill Adults’ (SWIPE) trial with more than 300 ICU patients without negative impact on kidney function [9], following previous evidence from observational studies [34]. The plasma-expanding effect of 20% IV albumin was compared in a small study of 15 hypoalbuminemic burn patients (serum concentration, 24.3 ± 4.7 g/L) with that in 15 healthy volunteers (serum albumin concentration, 38.3 ± 2.7 g/L); no significant differences were observed in the volume effectiveness of IV albumin [35]. Whether serum albumin concentration is an effect moderator of crystalloid-to-albumin fluid volume ratios in volume resuscitation remains poorly characterized.

### 3.1. Volume of Resuscitation Fluid Affected by Hypoalbuminemia in the Saline versus Albumin Fluid Evaluation (SAFE) Study

The SAFE study was conducted because, despite numerous clinical trials, it remained an open question whether the choice of IV solution in the fluid treatment of hemodynamic instability in ICU patients affects survival. In a double-blind RCT, heterogeneous patients received either 0.9% saline or 4% human albumin solution for IV fluid resuscitation for up to 28 days in the ICU, and all-cause mortality by day 28 was the primary endpoint. With 6997 patients participating, the study was large and informative because it was conducted at a low risk of bias. Both the primary endpoint and all secondary endpoints common in ICU studies were comparable between the two treatment groups. The main conclusion was that the two IV solutions led to similar outcomes in the indications studied [22].

Regarding the safety of 4% IV albumin in SAFE, it was concluded that, overall, there was no difference between the two IV solutions. In the subgroup of patients with traumatic brain injury (TBI), the treatment outcome with 4% albumin was worse than that with saline [36], but this was not related to albumin as a molecule but to the hypotonicity of the preparation used versus saline [37,38]. The recently published ‘Balanced Solution Versus Saline in Intensive Care Study’ (BaSICS) confirms this hypothesis. When comparing a balanced IV solution (Plasma Lyte 148^®^; Baxter Hospitalar, São Paulo, Brazil) versus isotonic saline, subgroup analysis of patients with TBI showed a significantly higher 90-day survival rate in the group randomized to isotonic saline than in patients treated with the balanced solution [39]. 

#### 3.1.1. Volumes of Study Fluids Administered in the SAFE Study

Patients in the SAFE study randomized to the albumin group received significantly lower study fluid volumes than those in the saline group, which, during the first ICU days, also resulted in significantly higher net fluid balance in the saline group than in the albumin group. The following ratios of the volume of albumin to the volume of saline administered were observed during the first four days: 1:1.3 on day 1, 1:1.6 on day 2, 1:1.3 on day 3, and 1:1.2 on day 4. The central venous pressure was significantly higher in the albumin group than in the saline group at all time points during the first four days [22].

#### 3.1.2. Effect of Baseline Serum Albumin Concentration on Outcomes

After the publication of the SAFE study results, another Cochrane meta-analysis confirmed that volume therapy with albumin compared with crystalloids did not affect mortality in a heterogeneous group of ICU patients; however, the possibility of an albumin-related mortality increase was still suggested for a selected group of patients with hypoalbuminemia [40].

Therefore, a post hoc analysis of SAFE determined whether the outcomes were influenced by baseline serum albumin concentration [41]. Data on baseline serum albumin concentration were available for 6045 patients; overall 2451 (40.6%) patients had a baseline serum albumin concentration of 25 g/L or less, and 3594 (59.4%) patients had a baseline serum albumin concentration of more than 25 g/L. Patients in the low baseline albumin concentration group were older, more likely to be admitted to the ICU after surgery, more likely to have severe sepsis or acute respiratory distress syndrome, and less likely to have had TBI. After adjusting for baseline risk factors for death, a baseline serum albumin concentration of 25 g/L or less was independently associated with the risk of death. The outcomes of resuscitation with albumin and saline were similar irrespective of the patients’ baseline serum albumin concentration. The results of this post hoc analysis confirmed that the administration of IV albumin is safe not only in heterogeneous ICU patients but also in ICU patients with hypoalbuminemia. Not only survival, but also secondary endpoints were similar in the two treatment groups with baseline serum albumin concentration of 25 g/L or less, including length of stay in the ICU, length of hospital stay, duration of mechanical ventilation, and duration of kidney replacement therapy [41].

#### 3.1.3. Effect of Baseline Serum Albumin Concentration on Ratios of the Volume of Albumin to the Volume of Saline Administered

Overall, in SAFE, a ratio during the first four days of approximately 1:1.4 was observed of the volume of albumin to the volume of saline administered [22]. Considering the volumes of study fluid used for hemodynamic stabilization in this double-blind pragmatic study, a significant difference between 4% albumin and 0.9% saline was observed mainly in the first ICU days (Table S1). In the patient group with baseline serum albumin concentration of 25 g/L or less, 4359 mL of IV saline and 2937 mL of IV albumin were administered as study fluids in the first seven ICU days (difference, 1422 mL); in the patient group with serum albumin of more than 25 g/L, these volumes were 3138 mL and 2661 mL, respectively (difference, 477 mL).

**Figure 4:**
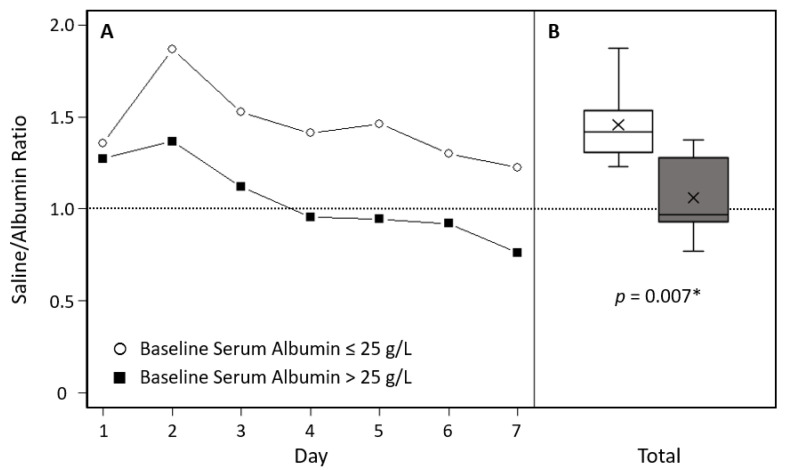
Ratios of the volume of albumin to the volume of saline by baseline serum albumin concentration of patients using data of the ‘Saline versus Albumin Fluid Evaluation’ (SAFE) Study. Data published in Ref. [41] were analyzed. (**A**) Mean saline to albumin ratios on days 1 to 7 in the two patient groups by serum albumin levels. (**B**) Box blots of mean saline to albumin ratios of days 1–7 in patients with baseline serum albumin concentration of 25 g/L or less and patients with serum albumin of more than 25 g/L. * Mann–Whitney U test.

The ratios of saline to albumin were not reported in the post hoc publication on the role of baseline hypoalbuminemia in SAFE and are therefore shown here. The mean resuscitation fluid volumes administered per day for the first seven days by the treatment group and baseline serum albumin concentration given in Ref. [41], as shown in Table S1, were used to calculate saline-to-albumin ratios. The mean saline-to-albumin ratio for days 1–7 was significantly higher in the group of patients with a baseline serum albumin concentration of 25 g/L or less than in the patient group with a serum albumin level of more than 25 g/L (Mann–Whitney U test n = 7, mean ± SD = 1.45 ± 0.211 vs. 1.05 ± 0.214, p = 0.007) (Figure 4). These data strongly suggest that the volume effects of IV albumin are likely to be stronger in hypoalbuminemia than in normal serum albumin levels.

### 3.2. Volume of Resuscitation Fluid in Randomized Controlled Trials on IV Albumin of Critically Ill Patients with Hypoalbuminemia

In an early ICU study by Dubois et al. [42], as part of their standard fluid management, patients randomized to the albumin group received 300 mL of 20% IV albumin on day 1 and 200 mL on subsequent days, provided that their serum albumin concentration was lower than 31 g/L; the control group patients received no albumin. Baseline serum albumin concentrations (mean ± standard deviation) in the albumin and control groups were 23.7± 3.7 and 23.2 ± 4.4 g/L, respectively; albumin serum levels did not change in the control group but increased rapidly in the albumin group from day 1 to 7. The mean daily fluid gain was almost three times higher in the control group than in the albumin group (1679 ± 1156 mL vs. 658 ± 1101 mL), although the use of diuretics was identical in both groups [42].

Against the background of plausible potential biomechanisms in sepsis [30] and the possible beneficial effect of IV albumin use in the SAFE study on a predefined subgroup of patients with sepsis [43], the ‘Albumin Replacement in Patients with Severe Sepsis or Septic Shock’ (ALBIOS) study investigated whether a systematic correction of decreased serum albumin levels with the use of IV 20% albumin affects the survival of patients with severe sepsis and septic shock [44]. Baseline serum albumin concentrations in the albumin and crystalloid groups were 24.1 ± 6.3 and 24.2 ± 6.2 g/L, respectively. Overall, the correction of low serum albumin levels had no effect on the survival of patients with severe sepsis or septic shock. Only a post hoc analysis suggested that the mortality of the non-stratified subgroup of patients with septic shock may be successfully improved, and this hypothesis is currently being prospectively studied in another RCT [45]. During the first seven days in ALBIOS, the median cumulative net fluid balance was significantly lower in the albumin group than in the crystalloid group (347 mL [interquartile range, −3266 to 4042] vs. 1220 mL [interquartile range, −2767 to 5034]) and the time to suspension of vasopressor or inotropic agent administration was shorter in the albumin group than in the crystalloid group [44].

### 3.3. Prevention of Intradialytic Hypotension in End-Stage Kidney Disease Patients with Hypoalbuminemia by Concentrated IV Albumin Solution

Patients with renal failure requiring dialysis often experience hypoalbuminemia, which is associated with adverse outcomes [46,47]. Intradialytic hypotension is more frequent in hypoalbuminemic patients [48] and is prognostically unfavorable, prolongs volume overload, and impairs volume reduction by dialysis. Fluid administration, including IV albumin, is a possible management modality [49].

Whether 5% IV albumin is more effective than 0.9% IV saline in the treatment of intradialytic hypotension was investigated in a double-blind, crossover RCT of 72 chronic hemodialysis patients [50]. Patients were randomized to either sequence–1 or sequence–2 of fluid choices, in which, when a hypotension episode occurred, either IV albumin was administered first, followed by IV saline for the second and third episodes, or IV saline was administered for the first episode, followed by IV albumin for the subsequent two episodes. Serum albumin concentrations in sequence–1 and sequence–2 patients were 38 ± 2 g/L and 38 ± 4 g/L, respectively. In these patients, relatively normoalbuminemic, chronic hemodialysis patients, 5% IV albumin was no more effective than 0.9% IV saline for the treatment of intradialytic hypotension [50].

In a small case series of eight patients with septic renal failure and renal replacement therapy published in the 1980s [51], intradialytic hypotension after priming with 0.9% saline was compared with 17.5% albumin priming; dialysis tolerance and left ventricular filling pressures were better after albumin priming. The administration of IV albumin to promote plasma refilling and to prevent intradialytic hypotension has been controversial for quite some time [52].

Recently, a post hoc analysis of the ‘Randomized Evaluation of Normal versus Augmented Level (RENAL) replacement therapy’ trial, in which more than half of the study participants received 4% or 20% IV albumin, demonstrated that a more negative fluid balance was achieved with 20% IV albumin than with 4% IV albumin, with no difference in mortality or renal recovery [53]. Plasma albumin levels in patients receiving only 4%, 20%, and 4% plus 20% IV albumin were 27 ± 7.3, 24 ± 6.4, and 24 ± 7.1 g/L, respectively. Administration of 20% IV albumin only resulted in a median (interquartile range) daily fluid balance of −288 mL (−904 to 261), whereas a positive daily fluid balance was recorded with 4% IV albumin only (245 mL [−248 to 1050]) or combined IV albumin (88 mL [−352 to 608]) (*p* < 0.001) [53].

Most recently, a relatively small study investigated whether prophylactic IV administration of 100 mL of 25% albumin compared with 100 mL of 0.9% saline was more effective in treating intradialytic hypotension and fluid removal in patients with acute renal failure or end-stage renal disease on dialysis whose serum albumin concentration was less than 30 g/L [54]. Serum albumin concentration before study fluid administration was 26.9 ± 3.4 g/L. Intradialytic hypotension after different definitions was reduced in the albumin group, and the ultrafiltration rate improved compared to that in the saline group [54]. 

## 4. Conclusions

Good-quality, meaningful RCTs of volume resuscitation show that administration of IV albumin results in lower net fluid balance than crystalloids. Prospective studies have shown that smaller infusion volumes may be sufficient for hemodynamic stabilization when IV albumin solutions are used instead of IV crystalloids. The achievable plasma volume expansion is greater with 20–25% than with 4–5% albumin solutions. In SAFE, the 0.9% saline to 4% albumin ratio of study fluid volumes was significantly higher in patients with a baseline serum albumin concentration of 25 g/L or less than in patients with a baseline serum albumin concentration of more than 25 g/L, suggesting that the lower the serum albumin level, the greater the volume effects of IV albumin. Correspondingly, the current best evidence suggests that IV administration of 20–25% human albumin solutions reduces the risk of intradialytic hypotension and improves the efficacy of renal replacement therapy in dialysis patients if serum albumin levels are decreased. Thus, clinical study data suggest that hypoalbuminemia acts as an effect moderator in volume resuscitation and plasma expansion with IV human albumin solutions. In clinical situations, the serum albumin level per se may inform when and how to include IV albumin in the need for large amounts of crystalloids in fluid management, which requires further studies.

## Supplementary Materials

The following supporting information can be downloaded at: https://www.mdpi.com/article/10.3390/ijms232214175/s1.
