# Supplementary material for: Moderator Effect of Hypoalbuminemia in Volume Resuscitation and Plasma Expansion with Intravenous Albumin Solution
Source: Int J Mol Sci. 2022 Nov 16;23(22):14175. doi: 10.3390/ijms232214175 (PMC9695189; doi:10.3390/ijms232214175)
Supplement: Supplementary file 1 [file ijms-23-14175-s001.zip › ijms-2034184-supplementary.pdf]

International Journal of *Molecular Sciences*

MDPI

Supplementary Material

Human Albumin Solution for Volume Resuscitation and Plasma Expansion in Patients with Hypoalbuminemia

Christian J. Wiedermann [1,2]

Table S1. Mean resuscitation fluid volumes administered per day for first seven days in intensive care in the Saline versus Albumin Fluid Evaluation (SAFE) Study by treatment group and baseline serum albumin concentration

Day	Baseline serum albumin ≤ 25 g/l					Baseline serum albumin > 25 g/l				
	N	0.9% saline per day (ml)*	N	4% albumin per day (ml)*	Saline-to-albumin ratio	N	0.9% saline per day (ml)*	N	4% albumin per day (ml)*	Saline-to-albumin ratio
1	1219	1650 (1585)	1221	1217 (981)	1.36	1808	1470 (1469)	1777	1154 (970)	1.27
2	1104	1211 (1745)	1093	648 (973)	1.87	1621	752 (1208)	1589	550 (803)	1.37
3	830	429 (911)	797	281 (554)	1.53	1158	290 (617)	1154	259 (554)	1.12
4	642	312 (846)	640	221 (449)	1.41	870	171 (456)	859	179 (420)	0.96
5	517	278 (674)	521	190 (416)	1.46	723	173 (463)	708	183 (448)	0.95
6	444	238 (617)	447	183 (447)	1.30	599	151 (486)	593	164 (428)	0.92
7	390	241 (682)	390	197 (474)	1.22	514	131 (381)	514	172 (469)	0.76

Resuscitation volumes administered because of hemodynamic instability are from Ref. [25] and were used for calculating the saline-to-albumin ratio. * Mean (standard deviation); N, number.
